# Endotheliotropic herpesvirus infection in Asian elephants (*Elephas maximus*) of Assam, India

**DOI:** 10.14202/vetworld.2019.1790-1796

**Published:** 2019-11-18

**Authors:** G. Mahato, K. K. Sarma, D. C. Pathak, N. N. Barman, P. Gogoi, M. Dutta, P. Basumatary

**Affiliations:** 1 Department of Veterinary Epidemiology and Preventive Medicine, College of Veterinary Science, Assam Agricultural University, Guwahati, Assam, India; 2 Department of Surgery and Radiology, College of Veterinary Science, Assam Agricultural University, Guwahati, Assam, India; 3 Department of Pathology, College of Veterinary Science, Assam Agricultural University, Guwahati, Assam, India; 4 Department of Microbiology, College of Veterinary Science, Assam Agricultural University, Guwahati, Assam, India; 5 Junior Research Fellow DBT-Twinning Project NER, College of Veterinary Science, Assam Agricultural University, Guwahati, Assam, India; 6 Department of Animal Biotechnology, College of Veterinary Science, Assam Agricultural University, Guwahati, Assam, India; 7 Assistant Manager, Centre for Wildlife Rehabilitation and Conservation, Bokakhat, Assam, India

**Keywords:** amplicons, amplification, elephant endotheliotropic herpesvirus, phylogenetic, polymerase chain reaction

## Abstract

**Background and Aim:**

Elephant endotheliotropic herpesvirus (EEHV) is an emerging disease of elephant. Therefore, a study was conducted to know the actual status of the disease in Assam State of India.

**Materials and Methods:**

A total of 289 Asian elephants of Assam were screened during 2 years of study from April 2017 to March 2019. The clinical symptoms of diseased as well as gross and histopathological changes of dead elephants were recorded for the diagnosis of the disease. Virus involved in the occurrence of the disease was confirmed by polymerase chain reaction (PCR).

**Results:**

In the present study, a total of three elephant calves out of 22 were found positive to EEHV1A. On the other hand, three adult asymptomatic elephants were also found positive for EEHV1 on screening 267 captive Asian elephants of Assam. The amplified PCR product showed band size of 520, 600, and 930 bp. The PCR amplified product with size 600 bp had shown the gene sequence for EEHV1U77/HEL. Gross lesions include congested blood vessels of the liver and intestinal mucosa, foci of petechiae in the spleen, and heart and focal ulceration in the dorsal surface of the tongue. Microscopically, the kidneys showed intertubular edema and focal areas of degeneration associated with coagulative necrosis of the tubular epithelium. The liver showed hydropic degeneration and fatty changes of the hepatocytes. There was a massive proliferation of fibroblasts in the interlobular spaces which penetrated the necrosed areas of the hepatic lobules.

**Conclusion:**

A total of three wild rescued elephant calves and three asymptomatic adults were found positive for EEHV1A during the 2 years of study. The PCR amplified product with size 600 bp had shown the gene sequence for EEHV1U77/HEL.

## Introduction

Elephant endotheliotropic herpesvirus-hemorrhagic disease (EEHV-HD) is a fatal disease of elephants caused by double-stranded DNA virus belonging to the subfamily *Betaherpesvirinae* under the genus Proboscivirus [1,2]. Till now, eight different genotypes of EEHV have been reported [3-6], of which EEHV 1A and EEHV 1B are considered to be the most common cause of high morbidity and mortality in captive Asian elephants [1,4]. The virus primarily affects elephant calves between 1 and 8 years of age, with a fatality rate of 80% [1,5,7,8]. The virus damages the inner lining of the small blood vessels, primarily the capillaries and is responsible for the rapid onset of acute hemorrhagic disease in Asian elephants. The disease is characterized by generalized edema of the head and limbs, oral ulceration, and cyanosis of tongue, trachea, and death within 7 days [9]. Deaths due to EEHV-associated disease cover approximately 65% of the overall mortality rate of captive-born Asian elephants in North America [10].

Incidence of EEHV is most commonly recorded in juvenile captive-born Asian elephants in North America [11]. In 2008 and 2014, only one lethal case had been reported from North America, while the incidence rate of the disease with high mortality had been observed in European zoos over the same time period [10].

In India, the incidence of EEHV-HD has also been reported, the first being in the year, 1997, and later on, 9 of 15 potential cases have been confirmed from Southern India in wild free-ranging calves in Kerala, Karnataka, Tamil Nadu forest reserves, and Madras Zoo [12]. A positive case of EEHV1A infection has also been reported from captive Asiatic elephants of Assam [13].

Every year several clinical cases have been recorded in captive and wild elephants suggested for EEHV infection; however, there is no systematic study on the disease in the northeastern region of India. An attempt has been made to investigate the prevalence of the disease in both captive and wild elephants in Assam.

## Materials and Methods

### Ethical approval

The present study was approved by the Department of Forest (Office of Chief Conservator of Forests, Wildlife & Chief Wildlife Warden, Assam), Government of Assam, to screen the rescued wild, departmental captive and private captive elephants of Assam.

### Study area

The study was undertaken at Kaziranga National Park, Orang National Park, and Pobitora Wildlife Sanctuary, Assam.

### Affected elephant

Blood and serum samples were collected from the elephant population under study following appropriate protocols [12]. Blood was collected in EDTA vials and in serum separator tube clot activator serum vials. A comprehensive postmortem procedure following elephant necropsy protocol was carried out within 24 h of death. Gross changes in the external body as well as in various internal organs were recorded. Tissue samples were collected and stored at −80°C until analysis. The samples were processed in 4-5 µ thick sections and stained with hematoxylin and eosin stains. The samples were subjected to molecular confirmation.

### Polymerase chain reaction (PCR) amplification

Standard operating procedure for the detection of EEHV was done as per the procedure described by Richman *et al*. [1], Latimer *et al*. [6], Barman *et al*. [13], Stanton *et al*. [14]. Three selected EEHV 1A gene loci representing U38/POL, U51/vG, and U77/HEL were targeted for PCR amplification. Briefly, the genomic DNA from suspected cases was extracted from blood, tissue, and serum samples (Nucleopore, Genetix brand). The PCR reactions were performed with a 25 µl total reaction volume resulting from 12.5 µl of master mix (Thermo Scientific), 1 µl of forward primer and reverse primer, 8.5 µl nuclease-free water, and 2 µl of DNA templates. The details of primer sets used are mentioned in Table-1. Thermal cycling conditions were as follows: Initial denaturation at 94°C for 5 min, denaturation at 94°C for 1 min, annealing at 56°C for 1 min, extension at 72°C for 1 min, and final extension at 72°C for 7 min with 36 cycles. The PCR products were electrophoresed in 1.7% agarose gel ethidium bromide in 1× Tris-acetate EDTA and visualized on ultraviolet transilluminator as per standard procedures. For size comparison, a 100 bp, DNA ladder marker (Thermo Scien­tific, USA) was run parallel to the PCR amplicons. The amplified PCR product was purified and sequenced by 1^st^ BASE DNA Sequencing, Malaysia.

**Table-1:** Details of primer sets used.

Gene	Primer sequence	Product size	References
EEHV Pan Pol./U38 PCR	5’-GTATTTGATTTYGCNAGYYTGTAYCC-3’ 5’-ACAAACACGCTGTCRGTRTCYCCRTA-3’	520 bp	[1,6,14]
EEHV1 U 51/vG PCR	5’-GATTGTGAACGCTGTAGTC-3’ 5’-GACTTTCTTCGTCGTAGCCCTCGTCTT-3’	930 bp	[1,6,14]
EEHV1 U77/HEL	5’-GCAAGGTRGAACGTATCGTCG-3’ 5’-CACAG[A/C]GCGTTGTAGAACC-3’	600 bp	[1,6,14]

EEHV: Elephant endotheliotropic herpesvirus-+

### Phylogenetic analysis

The phylogenetic analysis of the isolated EEHV genome sequence was done using the MEGAX software (Molecular Evolutionary Genetics Analysis Computer Software, Pennsylvania State University, USA). The presence of EEHV1A was confirmed from the blood and tissues of the calves and adult elephant by PCR.

## Results

A total of 22 elephant calves of 3 months-3 years of age were screened and three calves rescued from wild (orphan) were found positive for EEHV1A (Table-2).

**Table-2:** EEHV1A in wild and captive elephants of Assam.

Source	Calf/adult	Number of elephants examined	Affected	Clinical signs	PM findings	Histopathological changes	PCR result
Wild	Calf	10	3	Purplish /cyanotic discoloration and vesicles formation in tongue, swollen head, drooling of saliva, lateral recumbency	Congestion of liver, heart, spleen, and cecum	Severe intertubular edema of kidneys, proliferation of fibroblast cells in the liver, and loss of endothelial cells of blood vessels.	+ve
Captive	Calf	12	-	-	-	-	−ve
Captive	Adult	267	3	-	-	-	+ve

EEHV: Elephant endotheliotropic herpesvirus

**Figure-1:**
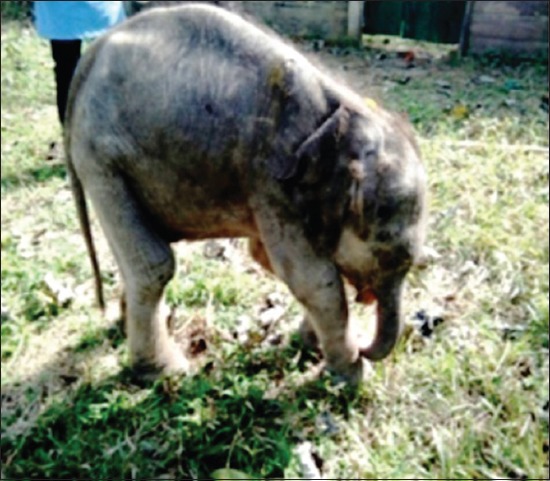
Swollen head.

**Figure-2:**
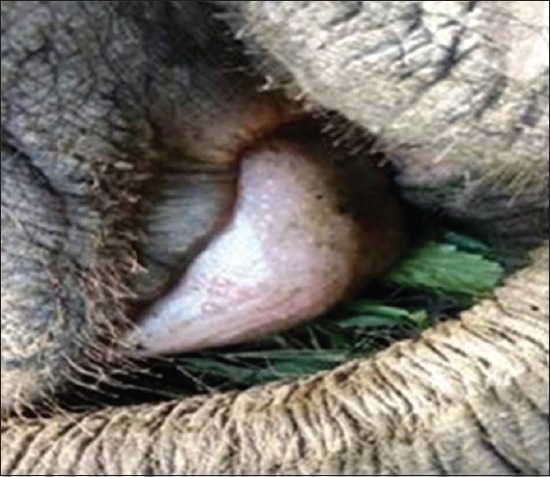
Edematous swelling with vesicles in tongue.

**Figure-3:**
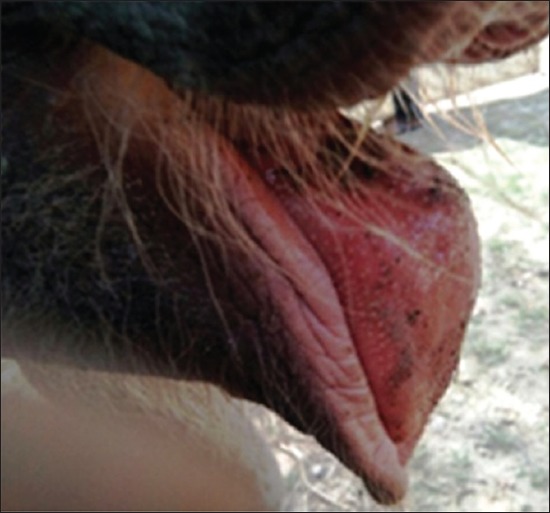
Inflamed mandibles.

**Figure-4:**
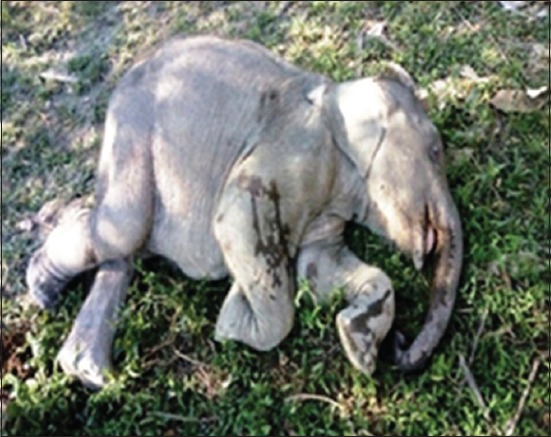
Lateral recumbency.

The elephant calves showed severe dehydration, incoordination, sleepy (lethargic) attitude, unable to stand (advanced stage), arched back condition, edema of the head region (Figure-1), purplish or cyanotic discoloration with edematous swelling of vesicles in tongue (Figure-2), swollen mandible (Figure-3) protrusion of tongue, and stringy thick saliva with neurological signs (one). There was an edematous swelling in the eyelids and mandibles with drooling of saliva accompanied by lethargy. The animal rejected solid food with frequent lying down and ultimately recumbency (Figure-4). Employing PCR in whole blood, tissue and serum samples, and EEHV1A was detected in the affected calves.

A total of 267 captive (Department of Forest, Government of Assam and private) elephants were also included in the study for a period of 2 years (December 2016-December 2018). The age ranged from 8 years to 75 years which lived primarily in captive situations of Kaziranga National Park, Orang National Park, Manas National Park, Nameri National Park, Pobitora Wildlife Sanctuary, Laokhowa Wildlife Sanctuary, and Garbhanga Wildlife Sanctuary that interacted extensively with free-ranging wild elephants. All the elephants included in this study were routinely under human care, allowed to enter the forest for foraging and patrolling duty. Screening results yielded only three adult elephants positive for EEHV1A, but none of the positive elephants exhibited any clinical signs and are still alive. One female elephant which was found positive is in advanced stage of pregnancy.

### Gross pathological findings

**Figure-5:**
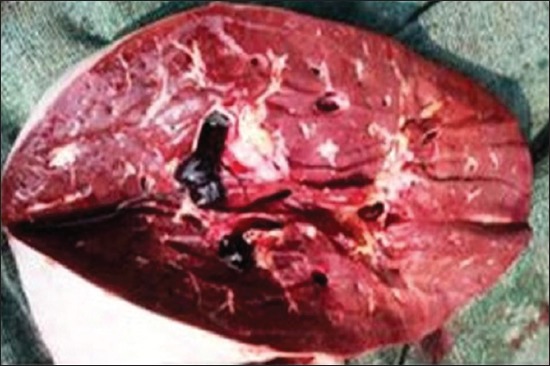
Congested blood vessels of liver.

**Figure-6:**
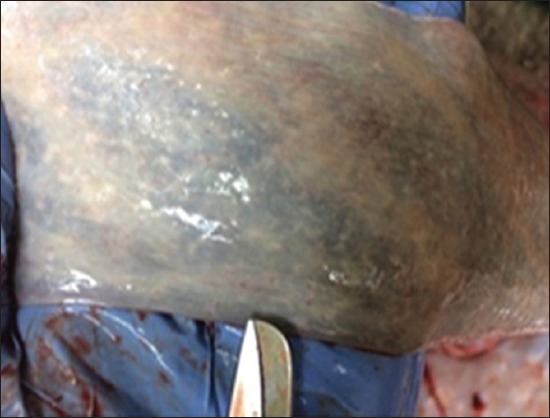
Petechial hemorrhage in spleen.

**Figure-7:**
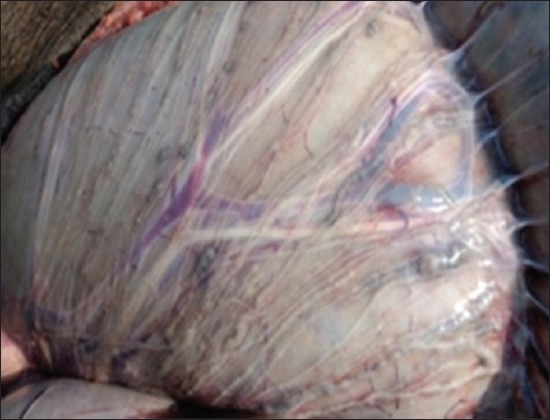
Congested serosal surface of stomach.

**Figure-8:**
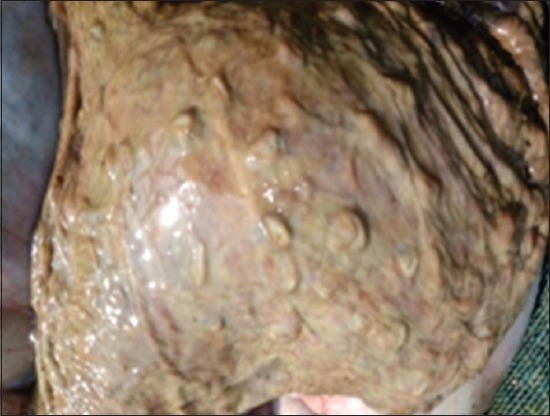
Congested mucosa of cecum.

Gross lesions seen in EEHV affected calves included congested blood vessels of the liver (Figure-5) catarrhal enteritis with congested intestinal mucosa, foci of petechiae in the spleen (Figure-6), myocardial hypertrophy with few epicardial petechial hemorrhages in the heart, mucosa of the cecum and serosal surface of the stomach (Figure-7), congested mucosa of the cecum (Figure-8), and focal ulceration in the dorsal surface of the tongue.

**Figure-9:**
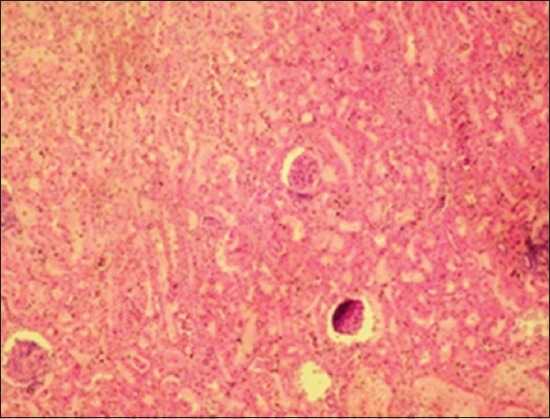
Severe intertubular edema in kidneys.

**Figure-10:**
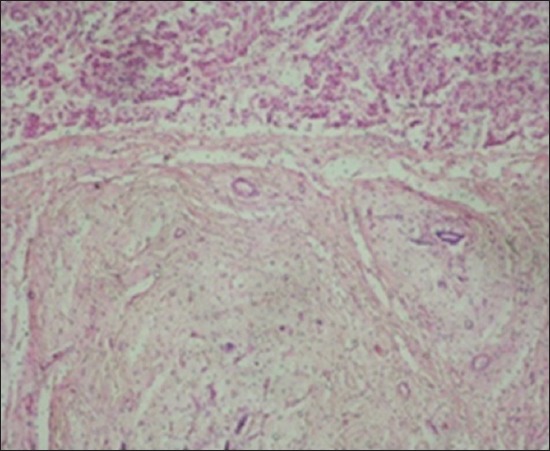
Proliferation of fibroblast cells replacing the necrotic hepatocytes in liver.

**Figure-11:**
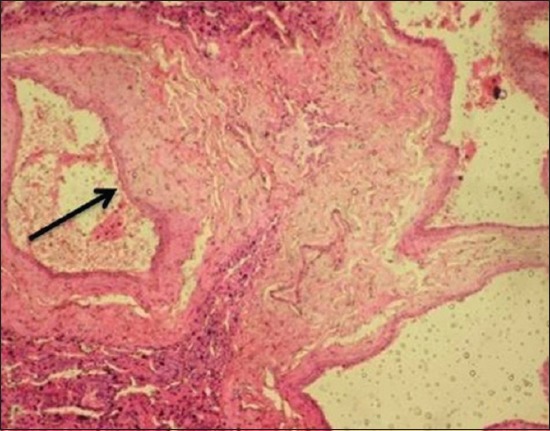
Blood vessels showing loss of endothelium.

**Figure-12:**
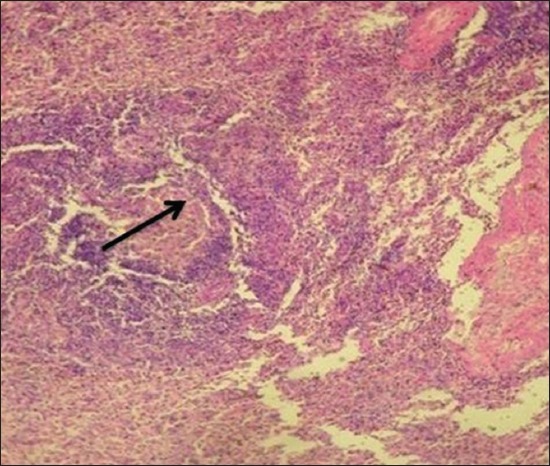
Depletion of lymphoid cells in the germinal center of the lymphoid follicles of the spleen.

Histopathological examination of the EEHV-infected elephant calf showed severe intertubular edema in kidneys (Figure-9) due to increase in capillary permeability due to possible damage to the capillary endothelium. There were focal areas of degeneration and coagulative necrosis of the tubular epithelium. Severe hydropic degeneration and fatty change of the hepatocytes were observed along with the massive proliferation of fibroblast cells replacing the necrotic hepatocytes (Figure-10). In the proliferated fibroma tissue, some biliary epithelial cells were seen intending to form new acini or biliary ducts. The empty capillaries were devoid of endothelium (Figure-11). Few congested capillaries with endothelium and partially hemolyzed erythrocytes with thickened vascular walls were observed. There was a depletion of lymphoid cells in the white pulp of the spleen (Figure-12).

### Molecular confirmation

**Figure-13:**
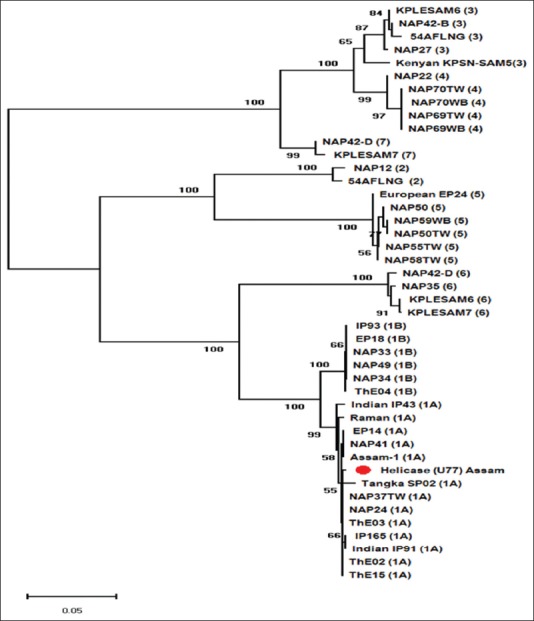
Phylogenetic tree of elephant endotheliotropic herpesvirus (EEHV) isolated from elephant of Assam based on helicase (U77) gene. The tree was constructed in MEGAX software by neighbor-joining method and substitution model used was Tamura 3-parameter as estimated to be the best fit model in MEGAX software on the basis of Bayesian information criterion (BIC). Different EEHV strains are represented in the figure. Numbers along the branches refer to the bootstrapping value (percentage of confidence). The partial helicase gene sequence (520 bp) of EEHV used in this study is highlighted with red solid circle and found to be clustered with EEHV1A group. EEHV1A was detected in three wild rescued elephant calves and three asymptomatic adult elephants of Assam during 2 years of study.

The presence of EEHV1A was confirmed in the blood and serum samples of the calves by PCR for three selected EEHV1A gene loci representing U38/POL, U51/vG, and U77/HEL. The amplified PCR product showed a band size of 520, 600, and 930 bp. Nucleotide sequence analysis of amplicons showed identity with the available EEHV sequences from the GenBank. Amplicons of 600 bp were sequenced and checked for similarities with known sequences with the Basic Local Alignment search tool (BLAST) algorithm of GenBank. The partial nucleotide sequences showed its identity with EEHV1A genome sequences on BLAST analysis. The PCR amplified product with size 600 bp had shown the gene sequence for EEHV1U77/HEL (NCBI – accession number MN207309). Phylogenetic analysis of polymerase, helicase, and GPCR genes showed clustering of the sequences with strains of EEHV1A (Figure-13).

## Discussion

PCR is the gold standard test for the detection of EEHV1A in elephants. The presence of other gamma herpesviruses cannot be ruled out for which the samples were further processed to detect new strain from the negative samples. In the study, three elephant calves and three asymptomatic adults were found positive to EEHV1. Similar reports of infection have also been reported from the southern part of India. [12]. EEHV1 has also been reported from the wild-born elephants which are in agreement with our findings because all the positive EEHV1 cases were wild-born calves [14]. Although there was evidence of EEHV in captive-born elephant calves [10,12], no positive case was recorded in the present study. The presence of EEHV1A strains in three adult asymptomatic healthy elephants was from the captive departmental elephants of Assam. PCR against EEHV3/4 and EEHV5 was performed in the blood sample of the EEHV1A-positive claves and adults, but none of the samples were found positive to have cross-infection with the other viral strains. A difficult management issue for EEHV disease is the short time frame between the appearance of clinical signs and death. However, several Asian elephants have survived severe EEHV-associated disease [1,12,14]. The survival of these animals was assisted in part by aggressive supportive therapies and administration of anti-herpes viral medications. The PCR assays will facilitate rapid and early diagnosis of potential disease, which would prompt initiation of treatment procedures known to have been associated with the increased rate of survival of other EEHV-infected elephants. In addition, the PCR assays could be used for regular monitoring of susceptible animals where one might even detect emerging viremia before the appearance of clinical signs.

## Conclusion

Out of 22 elephant calves screened, three wild rescued calves were found positive for EEHV1A. The disease has not been detected from captive-born calves. Of 267 adult captive elephants screened against EEHV1A, only three asymptomatic elephants were found positive during the 2 years of study. The PCR amplified product with size 600 bp had shown the gene sequence for EEHV1U77/HEL. No other strain of EEHV was detected from Assam.

## Authors’ Contributions

GM was involved in the study design, data collection, data analysis, conducting treatment, and writing of the manuscript while KKS and PB were involved in sample collection and therapeutic management. DCP interpreted the pathological alterations and NNB, PG, and MD were involved in molecular works. All authors read and approved the final manuscript.
